# Orientin and Cancer Suppression: Molecular Mechanisms and Synergistic Effects

**DOI:** 10.7150/jca.127661

**Published:** 2026-04-08

**Authors:** Emad A. Ahmed, Peramaiyan Rajendran

**Affiliations:** Department of Biological Sciences, College of Science, King Faisal University, Al-Ahsa, 31982, Saudi Arabia.

**Keywords:** orientin, cancer cell invasion and metastasis, anti-angiogenesis, combination treatments

## Abstract

Orientin, a C-glycosyl flavonoid (luteolin-8-C-glucoside) naturally present in plants such as *Ocimum sanctum* (holy basil), *Trollius chinensis*, rooibos tea, and *Passiflora* species, exhibits a wide range of pharmacological activities, including antioxidant, anti-inflammatory, and anticancer effects. In cancer research, orientin has been identified as a potent natural compound that can regulate several fundamental hallmarks of tumor progression, including excessive cell proliferation, resistance to apoptosis, angiogenic signaling, and metastatic dissemination. This review highlights orientin as a low-toxicity, naturally derived flavonoid that is generally well tolerated by healthy cells and may offer cytoprotective and antioxidant benefits, in contrast to conventional chemotherapeutics that often damage normal tissue, while selectively targeting cancer cells. The underlying molecular mechanisms responsible for orientin's inhibition of cancer progression, as well as its potential synergy with other therapies, are summarized. Collectively, these properties suggest a favorable pharmacological profile, supporting its consideration for clinical applications, especially in combination treatment approaches.

## Introduction

Orientin, a naturally occurring C-glycosyl flavone widely distributed in medicinal plants such as Ocimum sanctum (holy basil), Phyllostachys spp. (bamboo), and Passiflora incarnata, has emerged as a promising bioactive compound with diverse pharmacological properties. Among its many biological effects, increasing evidence highlights orientin's significant role in cancer prevention and therapy. As a multifunctional phytochemical, orientin exerts strong antioxidant, anti-inflammatory, and DNA-protective activities that contribute to maintaining cellular homeostasis 1-3. However, *in vitro* and *in vivo* studies demonstrated that orientin selectively targets malignant cells by modulating redox balance, inducing apoptosis, and suppressing tumor proliferation, invasion, and angiogenesis, while exhibiting minimal toxicity toward normal tissues^4^-^6^. Mechanistically, orientin acts through the regulation of key signaling pathways such as NF-κB, COX-2, Hedgehog, and HIF-1α/VEGF, thereby inhibiting tumor-promoting inflammation and angiogenic processes^7^, ^8^. Its dual role as both a cytoprotective antioxidant in normal cells and a pro-oxidant in cancer cells underscores its selective therapeutic potential.

Another structurally related compound to orientin is the isoorientin (ISO, luteolin-6-C-glucoside), a C-glycosyl flavone that has been recently reviewed for its effects on cancer cells proliferation, apoptosis, migration, and key signaling pathways across multiple *in vitro* cancer models, including lung, brain, oral, liver, pancreatic, and gastric cancers^9^. However, which has not been highlighted before, in this review we focus on orientin, a low-toxicity, naturally derived flavonoid that is generally well tolerated by healthy cells and may offer cytoprotective and antioxidant benefits but selectively targeting cancer cells. The underlying molecular mechanisms responsible for orientin's inhibition of cancer progression, as well as its potential synergy with other therapies, are summarized.

### Natural resources of orientin

Among natural sources, *Phyllostachys* spp. (bamboo) have consistently been reported as the richest repositories of orientin, where it can constitute a large fraction of the leaf flavonoid pool (≈4.9-7.8% of the total extractable flavonoid fraction) and, in some measurements, reach very high absolute levels, around 38mg/g dry leaf^1^, ^3^. *Passiflora* spp., particularly *Passiflora incarnata*, are also high-yielding sources (≈3.3 mg/g in some dried extracts) and commonly co-contain vicenin-2 and isoorientin, which together contribute to the pharmacological profile^10^. *Ocimum sanctum* (holy basil, *Tulsi*) is another well-documented medicinal source, in which orientin is among the dominant C-glycosyl flavonoids in leaves as identified by LC-MS/MS and HPLC profiling^2^. Other plant genera, including *Fagopyrum*, *Trollius*, *Adonis vernalis*, *Jatropha gossypifolia*, *Hordeum vulgare*, and *Anadenanthera*, typically contain moderate to low or trace amounts depending on plant part, cultivar, and extraction or analysis method 1, 3. Importantly, reported yields vary widely with analytical approach (HPTLC, HPLC, LC-MS), extraction solvent, plant part (young vs. mature leaves or sprouts), and environmental or developmental factors (light intensity, temperature, growth stage), all of which must be considered when comparing data across studies^1^, ^3^.

### Predicting the anticancer potential of orientin via *in silico* approaches

*In silico* studies have highlighted multiple molecular targets of orientin relevant to cancer therapy. Molecular docking analyses demonstrated that orientin interacts with matrix metalloproteinase-13 (MMP-13) - a promotor for tumor invasion, metastasis, and angiogenesis- by hydrogen bonding of orientin with Pro242 within the catalytic pocket, suggesting inhibition of substrate access and extracellular matrix degradation as well as potential inhibition tumor invasion^11^. These interactions suggest that the catalytic site of MMP-13 is a key targetable region, where orientin can potentially block extracellular matrix remodeling and metastasis. Additionally, orientin also demonstrated a high binding affinity for dual-specificity tyrosine phosphorylation-regulated kinase 2 (DYRK2). Molecular docking analyses suggested that it is stabilized within the kinase's ATP-binding site through hydrogen bonds and hydrophobic interactions, which could underlie its observed anti-proliferative activity in U87 glioblastoma and Caco-2 colorectal cell lines^12^. Binding to the ATP-binding pocket of DYRK2 implies potential modulation of downstream cell cycle regulators, including cyclin D1 and p53-mediated apoptotic pathways. Orientin also forms stable interactions near the ATP-binding pocket of JAK2, particularly at Lys882 and Asp939, residues essential for kinase catalysis, consistent with predicted inhibition of JAK2/STAT signaling, a central regulator of cell proliferation and survival 13-15. These docking results confirm the ATP-binding pocket and key catalytic residues of JAK2 as potential molecular targets for orientin. In the meantime, quinone oxidoreductase, a redox-regulating enzyme implicated in hepatocellular carcinoma, was predicted to accommodate orientin via hydrogen bonds at its catalytic site, potentially modulating oxidative stress and cytotoxic signaling^16^. Targeting quinone oxidoreductase may lead to regulation of ROS-dependent signaling pathways such as Nrf2 and MAPK, contributing to apoptosis and inhibition of tumor growth. However, nano-formulated orientin (NF-O) further improved predicted target engagement and bioavailability, enhancing interactions with angiogenesis-related proteins such as HIF-1α and VEGF, as indicated by stable binding to the PAS domain of HIF-1α, which may reduce tumor neovascularization^17^. The PAS domain of HIF-1α is a critical site for orientin binding in angiogenesis-related protein inhibition. These *in silico* findings suggest that orientin interacts with key hotspot residues across multiple cancer-related proteins, offering mechanistic insight into its multi-targeted anticancer activity and highlighting its potential for preclinical testing and future clinical application.

### Cytotoxicity of orientin in normal and cancer cells

Orientin was found to have selective cytotoxicity toward cancer cells while exerting minimal toxic effects on or even enhancing antioxidant capacity in normal cells, highlighting its potential as a safe and effective anticancer flavonoid. For instance, orientin was shown to have a minimal toxicity to NIH-3T3 fibroblasts (IC₅₀ >100 µM), consistent with a favorable safety margin in non-malignant cells and above 50 µM in H9c2 rat cardiomyocytes, indicating that orientin exerts its effects without noticeably compromising the viability or structural integrity of normal cells^18^. Similarly, orientin-treated normal epithelial and endothelial cells maintained normal proliferation and mitochondrial activity, suggesting a low risk of oxidative or apoptotic damage^19^. Moreover, recent cellular studies (e.g., keratinocyte and skin models; equine stromal cells) demonstrate orientin's protective effects against UV or oxidative stress and no evidence of harming normal cell viability at concentrations used for protection, reinforcing its low cytotoxic profile in normal cells 20, 21. In parallel with that, in human neuroblastoma SH-SY5Y cells, orientin was non-cytotoxic up to 20 µM and even enhanced antioxidant defense under hydrogen peroxide-induced stress, further confirming its biocompatibility^22^. Together, these observations support that orientin is well tolerated by normal cells and may exert cytoprotective or antioxidant effects, distinguishing it from conventional cytotoxic agents that often compromise normal tissue integrity 1, 23. On the other hand, *in vitro* studies have demonstrated that orientin significantly inhibits the proliferation of human cancer cell lines. These included hepatocellular carcinoma, colorectal carcinoma^4^, ^24^, ^25^, breast cancer^8^, ^26^, Colorectal (HCT-116) lung carcinoma (A549) cells, in a dose-dependent manner^5^. Moreover, in contrast, in multiple tumor models including colorectal, breast, and liver cancers, orientin triggered caspase-dependent apoptosis and mitochondrial dysfunction, hallmarks of selective cytotoxic action^27^. Overall, these results demonstrate that orientin exhibits selective cytotoxicity—potently targeting cancer cells while sparing normal ones—making it an attractive natural compound for anticancer therapy with a potentially wide therapeutic margin.

### Orientin promotes apoptosis in cancer cells

In EC-109 esophageal cancer cells, orientin induced dose-dependent apoptosis over 24-72 h. Colony formation assays showed increasing inhibition with concentrations from 5 to 80 µM. Flow cytometry analysis demonstrated that orientin at 20 µM, 40 µM, and 80 µM increased apoptotic rates progressively. In the meantime, morphological changes consistent with apoptosis, such as cell shrinkage, membrane blebbing, and nuclear condensation, were also observed at these doses^23^. In a study investigated the effects of 100 µM orientin on T24 human bladder carcinoma cells, orientin inhibited cell proliferation, induced cell cycle arrest, and promoted apoptosis via decreasing the expression of anti-apoptotic protein Bcl-2 and an increase in pro-apoptotic protein BAX and cleaved caspase-3. That was associated with the suppression of the NF-kappaB and Hedgehog signaling pathways^7^. However, treatment of HT29 human colorectal carcinoma cells with orientin resulted in a marked, dose-dependent induction of apoptosis accompanied by suppression of cell proliferation. Orientin exposure increased the proportion of apoptotic cells, with significant elevation of early and late apoptosis observed at 6.25 µM and 12.5 µM. At the molecular level, Orientin altered the BAX/BCL-2 ratio in favor of apoptosis via enhancing the mitochondrial release of cytochrome c and Smac/DIABLO into the cytosol, followed by activation of caspase-9 and caspase-3 and cleavage of PARP^25^. The study also reported DNA damage induction, evidenced by increased γH2AX expression, linking orientin's pro-apoptotic action to oxidative or genotoxic stress mechanisms. On the other hand, Flax straw, a byproduct of Linum usitatissimum L., contains high levels of C-glucosyl flavonoids, including orientin, vitexin, and isoorientin. Extracts from flax straw suppressed proliferation of MCF-7 cells and triggered apoptosis by increasing Bax and caspase-7, -8, and -9 expression, while decreasing Bcl-2 levels^26^. Conclusively and as summarized in table 2, orientin seems to induce the intrinsic apoptotic pathway by causing DNA damage, elevating oxidative stress, and impairing mitochondrial function, ultimately resulting in cytochrome c release and activation of caspases.

### Orientin inhibits proliferation, migration, and cell cycle progression in cancer cells

**Figure 1:**
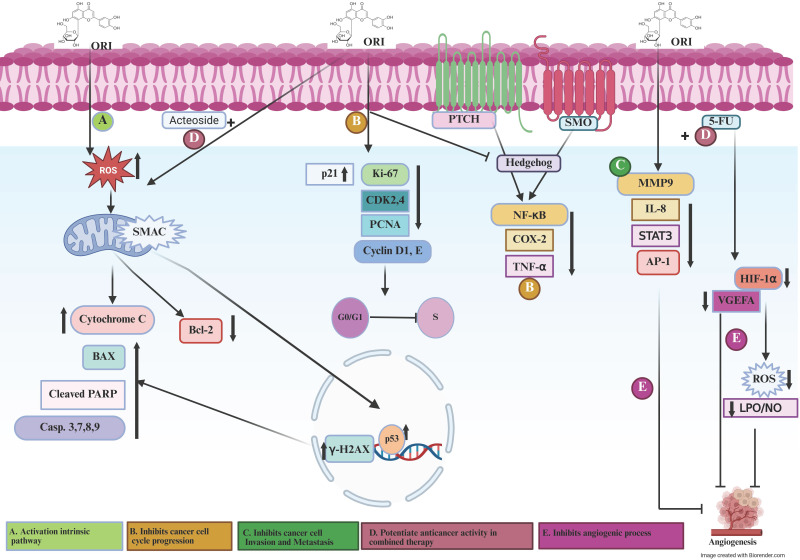
Signaling mechanisms involved in orientin preventing cancer progression. Figure 1. (A) Orientin activates the intrinsic apoptotic pathway by promoting DNA damage and intracellular ROS generation, leading to mitochondrial dysfunction, cytochrome c release, and subsequent caspase the migration activity. (B) Orientin inhibits cancer cell cycle progression, thereby suppressing the proliferative and migratory activities of cancer cells. (C) Orientin Inhibits Cancer Cell Invasion and Metastasis. (D) Orientin potentiates anticancer activity in combined Therapy. (E) Orientin inhibits angiogenic processes and regulates vascular dynamics. ORI, orientin; ROS, reactive oxygen species; SMAC/DIABLO, second mitochondria-derived activator of caspases/direct IAP-binding protein with low PI; Casp, caspase; PARP, poly (ADP-ribose) polymerase; BAX, Bcl-2-associated X protein; Bcl-2, B-cell lymphoma 2; p53, tumor suppressor protein p53; γ-H2AX, phosphorylated histone H2A.X; p21, cyclin-dependent kinase inhibitor 1; CDK2/4, cyclin-dependent kinase 2/4; Cyclin D1/E, cyclin D1/E; PCNA, proliferating cell nuclear antigen; PTCH, patched receptor; SMO, smoothened receptor; NF-κB, nuclear factor kappa B; COX-2, cyclooxygenase-2; TNF-α, tumor necrosis factor alpha; MMP-9, matrix metalloproteinase-9; IL-8, interleukin-8; PKCα, protein kinase C alpha; ERK, extracellular signal-regulated kinase; AP-1, activator protein 1; STAT3, signal transducer and activator of transcription 3; VEGFA, vascular endothelial growth factor A; HIF-1α, hypoxia-inducible factor 1 alpha; LPO, lipid peroxidation; NO, nitric oxide.

Orientin was found to exhibit potent antiproliferative and antimigratory activities in several cancer models (Figure 1). In hepatocellular carcinoma cells, Tao *et al*. reported that orientin significantly inhibited proliferation and migration of HepG2 and Huh7 cells in a dose-dependent manner^5^. This effect was accompanied by decreased levels of PCNA and MMP-2/9, suggesting disruption of DNA synthesis and extracellular matrix remodeling. In addition, orientin induced G0/G1 cell cycle arrest by downregulating cyclin D1 and CDK4 while upregulating the cell cycle inhibitor p21, indicating its potential to limit hepatoma growth by modulating the cyclin-CDK pathway and preventing entry into the S phase. Furthermore, orientin regulates members of the Bcl-2 family and inhibitors of apoptosis proteins, while also modulating the tumor suppressor p53, contributing to the activation of programmed cell death pathways^28^. In human bladder carcinoma T24 cells, Tian *et al*. demonstrated that orientin suppressed cell proliferation and promoted apoptosis by inhibiting the NF-κB and Hedgehog signaling pathways^7^. This effect was associated with downregulation of the anti-apoptotic protein Bcl-2 and upregulation of Bax and cleaved caspase-3, indicating interference with key survival and proliferative mechanisms. In the context of colorectal carcinogenesis, Thangaraj and Vaiyapuri demonstrated that orientin significantly suppressed colonic cell proliferation and mitotic activity in rats with 1,2-dimethylhydrazine (DMH)-induced colorectal cancer^4^. This antiproliferative effect was associated with downregulation of PCNA, Ki-67, and cyclin D1, alongside inhibition of NF-κB-mediated inflammatory mediators such as COX-2 and TNF-α, indicating that orientin modulates both inflammatory and cell cycle-regulatory pathways. Moreover, administration of orientin (10 mg/kg b.w., i.p.) during the initiation, post-initiation, or throughout the entire experimental period markedly reduced tumor marker levels and preserved near-normal colonic histoarchitecture compared to untreated controls. Altogether, these findings highlight orientin as a promising natural agent for colorectal cancer prevention and therapy through combined anti-proliferative and anti-inflammatory mechanisms^4^. Similarly, Thangaraj *et al*. confirmed that orientin mitigated DMH-induced colonic aberrant crypt foci and cell proliferation, reinforcing its preventive role in chemically induced colon tumorigenesis^24^. In esophageal cancer EC-109 cells, An *et al*. reported that orientin, along with its structural analogue vitexin, markedly inhibited cell proliferation and promoted apoptosis, effects that were associated with upregulated p53 expression and downregulation of the anti-apoptotic protein Bcl-2^29^. This modulation of p53-dependent signaling pathways contributes to orientin's cytostatic and pro-apoptotic activity. As a whole, these findings indicate that orientin exerts its antiproliferative and antimigratory effects by targeting multiple regulatory mechanisms, including NF-κB, Hedgehog, COX-2/PGE-2, and cyclin/CDK signaling pathways. By inducing G0/G1 arrest, downregulating MMPs and cyclins, and modulating apoptosis-related proteins, orientin demonstrates a multi-targeted approach to controlling tumor cell proliferation and migration across diverse cancer types.

### Orientin inhibits cancer cell invasion and metastasis

Orientin has demonstrated significant anti-metastatic properties across various cancer types by targeting key molecular pathways involved in cell invasion and metastasis. In TPA-stimulated MCF-7 breast cancer cells, Kim *et al*. reported that orientin suppressed migration and invasion by downregulating MMP-9 and IL-8 expression through the PKCα/ERK/AP-1/STAT3 signaling pathway, which is critical for extracellular matrix remodeling and metastatic behavior^8^. Furthermore, Ghosh *et al*. demonstrated that orientin, when combined with 5-fluorouracil (5-FU), significantly reduced cancer stem cell (CSC)-mediated angiogenesis and metastasis in colorectal cancer by suppressing HIF1α and VEGFA expression^6^. Elumalai *et al*. reported that orientin inhibited proliferation and migration in human liver cancer cell lines, highlighting its potential as a therapeutic agent against cancer metastasis^17^. Collectively, these findings emphasize orientin's multifaceted ability to suppress cancer cell invasion and metastatic progression, supporting its promise as a candidate for developing anti-metastatic therapies.

### Synergistic anticancer effects of orientin in combined therapy

**Figure 2:**
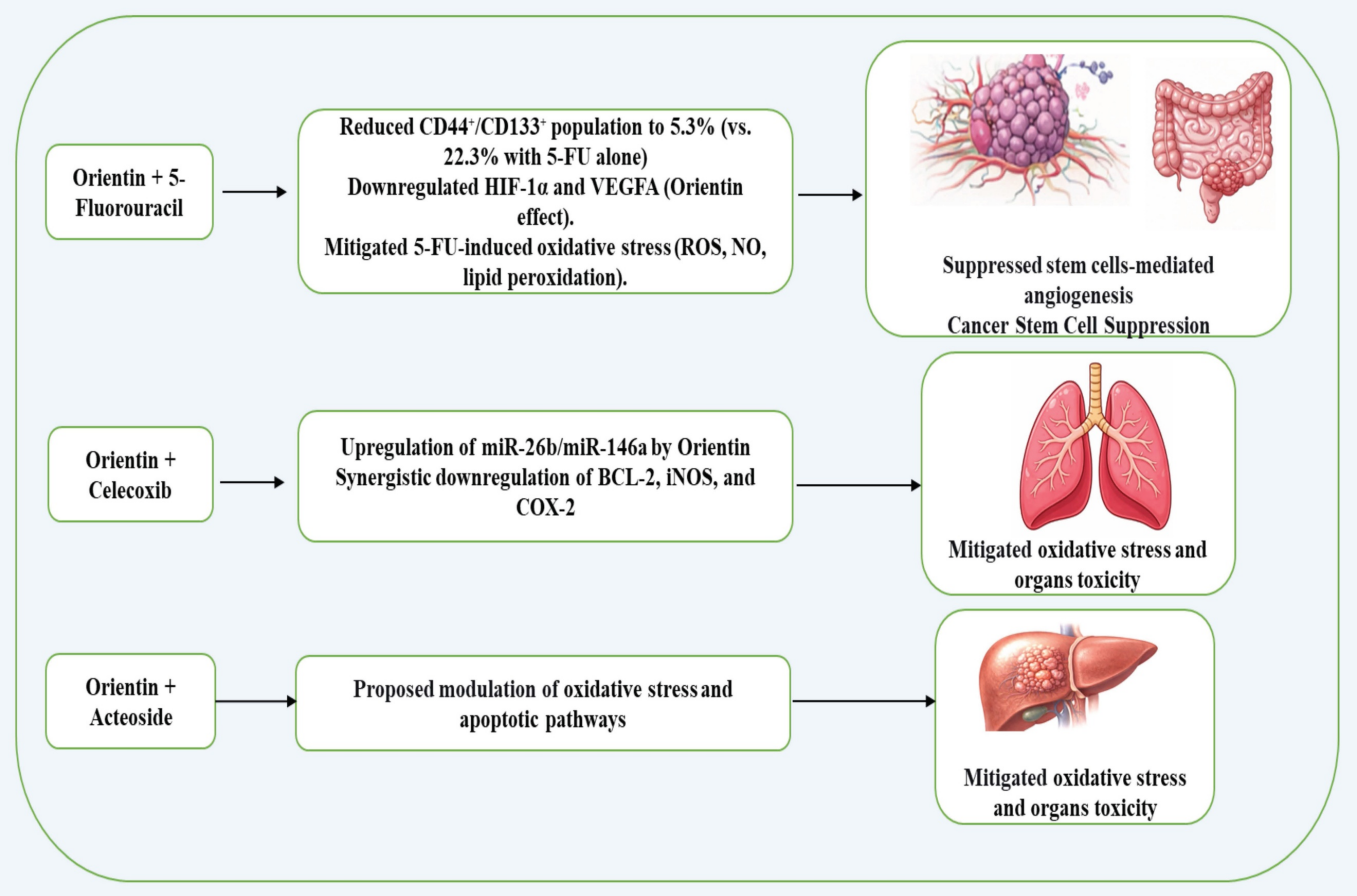
Possible synergistic anticancer effects of orientin in combined therapy.

When being combined with 5-fluorouracil in colorectal cancer, orientin potentiated the 5-fluorouracil effect by suppressing cancer stem cell-mediated angiogenesis through downregulation of HIF-1α and VEGFA, while also mitigating 5-fluorouracil-induced oxidative stress and organ toxicities^6^. In this combination treatment, orientin markedly decreased the cancer stem cell (CSC) population (CD44⁺/CD133⁺) in HCT116-derived tumorospheres from 22.3% with 5-FU alone to 5.3% with the combination, indicating a strong synergistic effect. That combination also significantly suppressed reactive oxygen species (ROS), nitric oxide (NO), and lipid peroxidation, key mediators of oxidative stress and angiogenic signaling^6^. Additionally, Orientin has been reported to synergize with the natural compound, Acteoside, to enhance anticancer effects via reduction of oxidative stress and modulation of apoptotic pathways^30^. Moreover, Khalil *et al*. reported that orientin synergizes with the COX-2 inhibitor celecoxib to more effectively inhibit A549 lung cancer cell migration, invasion, and pro-survival signaling than either agent alone, indicating a potentiated anticancer effect of the combination treatment^18^. In all, these studies (summarized in Figure 2) indicate that orientin can act as a synergistic agent in cancer therapy, enhancing the efficacy of existing treatments while reducing toxicity and limiting tumor progression.

### Orientin's anti-angiogenic and vascular-regulating potential

In colorectal cancer, orientin demonstrates strong anti-angiogenic activity by targeting CSC-driven and endothelial-mediated vascularization pathways (Ghosh *et al*., 2025). Interestingly, conditioned media from orientin-treated tumorospheres inhibited HUVEC tube formation and sprout elongation in chick chorioallantoic membrane (CAM) assays compared with 5-FU alone, confirming an anti-angiogenic effect. Furthermore, nano-formulated orientin (NF-O) enhanced these effects by improving bioavailability, reducing endothelial proliferation markers (CD31), and inhibiting VEGFR phosphorylation, underscoring its potential as a synergistic adjuvant to 5-FU in targeting CSCs and angiogenesis^17^. Beyond oncology, orientin modulated angiogenesis in vascular injury and reparative contexts where it reduced vascular smooth muscle cell proliferation, inflammatory infiltration, and vessel wall thickening in angiotensin II-induced injury^31^, but promoted angiogenesis in diabetic wound healing by enhancing tube formation and upregulating VEGF and FGF-2 via activation of the Nrf2/GPX4 pathway and reduction of lipid peroxidation^32^. Overall, orientin acts as a context-dependent vascular modulator—strongly inhibiting pathological angiogenesis in tumors while promoting functional neovascularization in tissue repair—highlighting its versatility as a therapeutic agent for oncology and regenerative medicine. Taken together, these findings indicate that orientin functions as a context-dependent vascular modulator, suppressing angiogenesis in tumors and pathological remodeling while promoting vascular regeneration during wound healing.

### Orientin in medicinal plant extracts and its anticancer potential

In studies dedicated to investigating the anticancer effects of flavonoids in medicinal plant extracts, orientin has been identified among other bioactive compounds as contributing to cytotoxic effects against cancer cell lines. In *Passiflora mucronata* leaf extract, HPLC-MS analysis detected orientin alongside isoorientin, vitexin, and other flavones, and the hexane fraction exhibited notable cytotoxicity, suggesting that these flavonoids may contribute to apoptosis induction and inhibition of tumor cell proliferation^33^. Similarly, the extract of flax straw, a byproduct of *Linum usitatissimum* L., contained orientin, vitexin, and isoorientin, suppressed proliferation of MCF-7 breast cancer cells by inducing apoptosis, increasing Bax and caspase-7, -8, and -9 expression, and decreasing Bcl-2 levels^26^. In addition, a SIRT6 “fishing” approach using *Trigonella foenum-graecum* seed extract identified orientin as a binder of the histone deacetylase SIRT6, demonstrating its interaction with biologically relevant targets within complex plant matrices^34^. Together these findings highlight orientin as a bioactive phytochemical in medicinal plant extracts with promising anticancer effects, supporting its further investigation in natural product-based cancer research.

Based on the available data to date, orientin exhibits a broad spectrum of anticancer activities, including induction of apoptosis, inhibition of proliferation, migration, and cell cycle progression, and suppression of invasion, metastasis, and angiogenesis. Furthermore, its synergistic interactions with conventional chemotherapeutics highlight its potential to enhance treatment efficacy by modulating key oncogenic signaling pathways. A detailed mechanistic overview of orientin-mediated molecular pathways involved in cancer is illustrated in Table 2 and Figure 1. (A) Activate the intrinsic apoptotic pathway via inducing DNA damage and stimulating intracellular ROS production, causing mitochondrial dysfunction and subsequent release of cytochrome c. This cascade triggers caspase activation and promotes PARP cleavage, culminating in apoptosis, and involves BAX and Bcl-2 (pro- and anti-apoptotic regulators of mitochondrial integrity), p53 (DNA damage-responsive transcription factor), γ-H2AX (marker of DNA double-strand breaks), and SMAC/DIABLO (pro-apoptotic mitochondrial protein that neutralizes inhibitor of apoptosis proteins, IAPs). (B) Suppress cell cycle progression through disrupting of key checkpoints controlling the G1/S transition by downregulating CDK2/4 and Cyclin D1/E (cell cycle drivers) and upregulating p21 and PCNA (cell cycle inhibitors and repair proteins), thereby restricting uncontrolled proliferation and migration of cancer cells. (C) Inhibit cancer cell invasion and metastasis and thus attenuates the invasive and metastatic potential of cancer cells by modulating pathways that regulate extracellular matrix degradation and cytoskeletal remodeling, limiting cell motility and metastatic spread**.** (D) Potentiate anticancer activity in combination therapy by enhancing the efficacy of co-administered chemotherapeutic agents, such as 5-FU and acteoside, through promoting anti-angiogenic effects, sensitizing tumor cells to apoptosis, and reducing drug resistance, thereby indicating synergistic potential. (E) Inhibit angiogenesis and regulate vascular dynamics via disrupting angiogenic signaling by downregulating VEGFA and HIF-1α (key mediators of vascular growth under hypoxia) and modulates inflammatory and oxidative cascades involving NF-κB (master regulator of cytokine expression), COX-2 (prostaglandin biosynthesis enzyme), TNF-α and IL-8 (proinflammatory cytokines), MMP-9 (extracellular matrix-degrading protease), PKCα and ERK (kinases that drive proliferation and survival), AP-1 and STAT3 (transcription factors regulating invasion and angiogenesis), as well as oxidative mediators such as LPO and NO. Additionally, orientin interferes with Hedgehog signaling through PTCH and SMO, thereby inhibiting tumor-associated angiogenic and proliferative responses.

## Figures and Tables

**Table 1:** Orientin promotes apoptosis in cancer cells

Cell Line / Cancer Model	Orientin Conc. / Treatment	Apoptotic Markers / Pathways Affected	References
Esophageal cancer (EC-109)	80 µM	↑ p53; ↓ Bcl-2	An *et al*., 2015
Human bladder carcinoma (T24)	20-100 µM	↓ Bcl-2; ↑ Bax and cleaved caspase-3	Tian *et al*., 2019
Human colorectal carcinoma (HT-29)	6.25-12.5 µM for 24 h (IC50 ≈ 6 µM)	↑ Bax; ↓ Bcl-2; cytochrome c & Smac/DIABLO release; ↑ cleaved caspase-9, caspase-3, PARP; ↓ XIAP, survivin; ↑ p53, γH2AX; indicates intrinsic mitochondrial apoptosis	Thangaraj *et al*., 2019
Human bladder carcinoma (T24)	10-40 µM for 24-48 h	Morphological apoptosis: nuclear condensation, DNA fragmentation; increased apoptotic index (Annexin V/PI staining)	Shen *et al*., 2020

**Table 2:** Comprehensive summary of orientin's anticancer mechanisms

Cancer Type / System	Mechanism of Action	Model System	Reference
Colorectal Cancer	Inhibits angiogenesis via HIF-1α/VEGFA suppression; attenuates NF-κB-mediated inflammation	*In vitro* (cancer stem cells, CSCs); *in vivo* (DMH-induced rat model)	Ghosh *et al*. (2025); Thangaraj & Vaiyapuri (2017)
Esophageal Cancer	Induces apoptosis via mitochondrial pathway activation	EC-109 cell line	An *et al*. (2015)
Hepatocellular Carcinoma	Inhibits proliferation and migration; modulates EMT markers	HepG2 cells; xenograft models	Tao *et al*. (2023)
Breast Cancer	Suppresses invasion by downregulating MMP-9 and IL-8 via PKCα/ERK/AP-1/STAT3 signaling	MCF-7 cells (TPA-induced)	Kim *et al*. (2018)
Liver Cancer	Exhibits cytotoxic activity; C-glycoside moiety identified as pharmacophore	HepG2 cell line	Sharma *et al*. (2016)
Bladder Cancer	Triggers apoptosis through inhibition of NF-κB and Hedgehog signaling pathways	T24 cell line	Tian *et al*. (2019)
Vascular Endothelium	Reduces oxidative stress and inflammation by enhancing SESN1-mediated autophagy	HUVECs (ox-LDL-induced model)	Gao *et al*. (2024)
Radiation Protection	Protects lymphocytes and bone marrow cells by minimizing chromosomal damage	*In vitro* (human lymphocytes); mouse models	Vrinda & Uma Devi (2001); Nayak & Devi (2005)
Chemosensitization	Enhances the efficacy of 5-FU in colorectal CSCs	*In vitro* and *in vivo* models	Ghosh *et al*. (2025)

## Data Availability

Data of the present study will be provided on request.
